# Reading subtyping of Arabic-speaking university students: a contribution to the accuracy vs. rate model of dyslexia

**DOI:** 10.1007/s11881-025-00323-4

**Published:** 2025-02-13

**Authors:** Bahaa Madi Tarabya, Samer Andria, Asaid Khateb

**Affiliations:** 1 The Unit for the Study of Arabic Language, Edmond J. Safra Brain Research Center for the Study of Learning Disabilities, University of Haifa, 199 Abba Khoushy Ave, Mount Carmel, Israel; 2 Dept of Learning Disabilities, Faculty of Education, University of Haifa, Haifa, Israel

**Keywords:** Arabic, Dyslexia, Reading accuracy, Reading rate, Subtypes

## Abstract

The current study sought to examine the existence of reading subtypes based on specific accuracy and rate criteria in dyslexia among a non-clinical sample of 120 Arabic-speaking University students and to characterize their reading-related and linguistic skills. For this aim, we relied on a conventional practice in reading disability literature based on a 25th percentile low achievement cut-offs. All students were examined using a battery of reading, linguistic, and cognitive tasks. The analysis conducted here provided partial support for the validity of the subtyping approach based on the accuracy and rate achievements and showed that ~ 12.5% of the sample could be defined as low accuracy (LA), ~ 10.8% as low rate (LR), and ~ 10.8% as a combined doubly low subtype (DL). The results showed that the combined DL group exhibited the lowest scores in all reading-related and linguistic tasks. Also, specific response patterns were found for LA and LR subtypes in reading-related pseudowords’ decoding and phonological awareness measures. These findings, the first to provide insights into the question of adult dyslexia through analysis of reading subtypes among Arabic-speaking students, are discussed in relation to previous findings in the literature among speakers of other languages. The data presented here provide new support for the adequacy of the accuracy vs. rate model of dyslexia among adults and stress the need for developing different interventions for the different reading subtypes.

**Supplementary Information:**

The online version contains supplementary material available at 10.1007/s11881-025-00323-4.

## Introduction

Developmental dyslexia is a disorder characterized by difficulties with accurate and/or fluent word recognition and poor spelling abilities. Typically diagnosed in childhood, these difficulties may persist into adulthood (Gerber, 2012; Pammer, 2014). Approximately 5–17% of school-age children (Erbeli et al., 2021; Fletcher et al., 2018; Wagner et al., 2020; Yang et al., 2022) and 4–5% of adults (Elsherif et al., 2021; Shaywitz & Shaywitz, 2005) can be found with this condition. Dyslexia is considered as a specific learning disability with a neurobiological origin, but also might be influenced by environmental factors (Erbeli et al., 2021; Grigorenko et al., 2020; Tannock, 2014). Despite adequate instruction and intelligence, individuals with dyslexia often struggle with accurate and fluent word recognition and spelling (Peterson & Pennington, 2012; Roitsch & Watson, 2019), which is generally attributed to a phonological processing deficit that is often unexpected in relation to the other cognitive abilities (Shaywitz & Shaywitz, 2020). The consequences of dyslexia may include difficulties in reading comprehension and a reduced reading experience, both of which can impede the growth of vocabulary and background knowledge (Lyon et al., 2003).

Although the relationship between phonological awareness and reading has been a subject of considerable debate (Share, 2021), there is still a consensus among researchers that phonological impairment underlies many specific reading difficulties (Jorm & Share, 1983; Shankweiler & Liberman, 1989; Snowling et al., 2020; Stanovich, 2000; Wagner & Torgesen, 1987). However, some researchers argue that the phonological deficit hypothesis cannot fully explain all reading disabilities (Nation & Snowling, 2004; Share, 1995). Consequently, a deficit in rapid automatized naming (RAN) as a domain-specific speed of processing impairment has been proposed as an additional risk factor for dyslexia (see also Pennington et al., 2001; Wolf & Bowers, 1999). Evidence for the role of RAN in the variance of dyslexia has been demonstrated across various languages (Catts et al., 2002; Fawcett & Nicolson, 1994; Fawcett et al., 1996; Sobotka & May, 1977; Stanovich, 2000; Willcutt et al., 2005). These observations have led to the proposition that reading disability may originate from different underlying sources (Pennington, 2006; Peterson & Pennington, 2012; Ramus et al., 2003) and to attempts to define specific subtypes.

Castles and Coltheart (1993)’s dual-route model was one of the first to subdivide reading disorders based on acquired reading difficulties among English speakers. According to this model, two routes exist for reading: (i) the non-lexical/sub-lexical route which involves reading new words or non-words through decoding and (ii) the lexical route through which a word as whole is retrieved and is involved in reading frequent or irregular words. In particular, the authors suggested that phonological dyslexia involves a selective impairment of the non-lexical route, while surface dyslexia results from an impairment of the lexical route. This distinction, primarily based on English, a language with a deep orthographic system and irregular words, has been questioned (Share 2008) given that many alphabetic writing systems are characterized by highly regular grapheme-phoneme conversion rules, where reading accuracy is not the primary issue. Indeed, evidence suggests that in these languages, reading accuracy improves rapidly, often reaching a ceiling effect by the end of first grade (Ziegler & Goswami, 2005). In such cases, fluency becomes the discriminating measure of individual developmental differences. Examples of these cases, where most dyslexics attain high levels of reading accuracy but remain slow, are provided in different languages including German (Wimmer, 1993), Italian (Paap & Noel, 1991), Greek (Porpodas, 2013), and Hebrew.

Therefore, some authors have attempted to conceptualize reading disability in terms of accuracy-based versus rate-based impairment (Leinonen et al., 2001; Lovett, 1984a, 1987; Shany & Share, 2011). Early on, Lovett (1984a, 1984b, 1987) categorized a clinical sample of children (aged 8 to 13) as either accuracy-disabled or rate-disabled, comparing them on various measures of oral and written language, based on multiple measures of decoding accuracy and rate. Those in the accuracy-disabled group also exhibited impairments in reading rate, making them slower readers compared to those in the rate-disabled group. Thus, the accuracy-disabled group was more severely impaired, showing a “double-disability,” whereas the rate-disability subgroup exhibited only a “single disability.” Later, Leinonen et al. (2001) classified Finnish adults with dyslexia into two subgroups based on the speed and accuracy of their text reading: the so called “*hasty*” and “*hesitant*” subgroups. *Hasty* dyslexic readers read quickly but were less accurate in oral text reading and phonological decoding tasks. In contrast, the *hesitant* dyslexic readers performed more accurately on all reading tasks than hasty readers but read more slowly. In parallel, Wolf and Bowers (Wolf & Bowers, 1999, 2000) acknowledged the crucial role of phonological awareness in learning to read and proposed that RAN is a second independent core deficit in dyslexia. The recognition of PA and RAN as independent sources of reading failure led to the development of the double-deficit hypothesis, which categorizes readers based either on one single deficit (i.e., *PA deficit or RAN deficit*) or on a double-deficit (i.e., *PA* + *RAN deficits*) (Wolf & Bowers, 1999, 2000). Numerous studies on reading in English and other languages with more shallow orthographies have found that RAN is more strongly related to reading fluency than reading accuracy, while phonological awareness was more strongly related to reading accuracy than reading fluency (see for a review Norton & Wolf, 2012). In line with this observation, a similar pattern of results was described in a cross-sectional study of Arabic-speaking first-to-sixth grade children (Asadi et al., 2017). This study investigated the contribution of linguistic and cognitive factors to decoding accuracy and fluency and found that while PA consistently explained variance in accuracy across all grades, RAN was a significant and consistent predictor of decoding fluency. In a subsequent study of Arabic-speaking third and fourth graders, the double-deficit theory of dyslexia was validated based on PA and RAN scores among a large sample of children with reading difficulties. The study identified three dyslexic groups: two groups presenting one single deficit either in PA or in RAN and one having a double deficit in both PA and RAN. The authors also reported that differences between the groups extended to reading, and to other cognitive, and linguistic abilities (Asadi & Shany, 2018).

Using another subtyping approach based on measures of reading accuracy and reading rate, a series of studies conducted on Hebrew and Arabic readers by Shany and colleagues (Shany & Breznitz, 2011; Shany & Share, 2011; Shany et al., 2023) provided evidence for a true double dissociation between accuracy-based and rate-based dyslexic subtypes. In their first investigation on Hebrew-speaking children (Shany & Breznitz, 2011), the authors sought to identify children who exhibited impairment in only one dimension of reading while maintaining normal levels of performance in the other dimension, using a large sample of Hebrew-speaking fourth graders. For this purpose, they relied on a conventional method in the literature using a 25th percentile low achievement cut-off (Siegel, 1999). They identified a specific accuracy-impaired subgroup and an equally specific rate-impaired subgroup, finding that these subgroups performed distinctively on variables other than the measures used to define them. The rate-impaired subgroup showed a slow RAN while the accuracy-only subgroup exhibited a selective deficit in phonological awareness. Also, the study showed that the doubly-disabled (< 25th percentile in both accuracy and rate) subgroup constituted the most severely impaired readers. Also, Shany and Breznitz (2011) analyzed data from a large clinical sample of adult university students. They found dissociation: with the accuracy-disabled subgroup displaying impaired accuracy with normal reading rate, while the rate-disabled subgroup exhibited a deficit in terms of reading rate but with normal accuracy. In a recent replication of Shany and Share’s findings, Shany et al. (2023) analyzed data from Arabic speaking fourth graders using the same criteria (i.e., scores ≤ the 25th and of ≥ 35th percentile) on measures of reading accuracy and reading rate and reported a clear double dissociation between groups. The authors also reported that differences between the subgroups extended to other linguistic and cognitive processes beyond PA and naming speed abilities.

Although research involving children is essential for understanding the development of early reading skills and the cognitive abilities that predict reading success, it provides limited insights into the long-term persistence of reading challenges and the reading-related and linguistic skills of adults in higher education (Reis et al., 2020). Despite the persistence of reading difficulties into adulthood, the cognitive abilities of adults with reading difficulties have been less explored than those of children. Given the significant impact of adults’ reading performance on academic achievements in higher education, understanding and characterizing their reading-related and linguistic skills are crucial. Previous research on dyslexia among higher education students has been conducted on heterogeneous groups of dyslexic readers with varying cognitive abilities (see Bekebrede et al., 2010; Faísca et al., 2023; Heim et al., 2008; Zoubrinetzky et al., 2014). In one early study, Hatcher et al. (2002) sought to identify the strengths and weaknesses of dyslexic adults. They examined 23 university students with dyslexia and 50 controls using 17 tasks, including reading, writing tasks, processing skills (perceptual speed, memory span, and arithmetic), phonological skills, verbal fluency, and verbal abilities. Their results indicated that the dyslexic group performed worse on all tasks, with significant deficits in reading, writing, and in reading-related phonological processes, as well as slower processing speed and reduced short-term memory spans (Hatcher et al., 2002). In the same vein, Swanson and Hsieh (2009)’s meta-analysis of 52 published articles confirmed these findings about adult dyslexics’ abilities, in particular underlining, not surprisingly, that the top problems of adults with dyslexia are writing, reading, and phonological processing (Swanson & Hsieh, 2009).

A significant body of research on adult dyslexia has focused on English, a language with a very opaque orthography. However, a recent meta-analysis carried out by Reis et al. (2020) proposed that orthographic transparency is an important factor affecting the manifestation of dyslexia symptoms. This meta-analysis indicated that adult dyslexia may present less prominently in languages with more transparent orthographies and that in such orthographies, PA is a minor problem. Miller‐Shaul (2005) compared the performance of adult dyslexics (diagnosed either in childhood or upon entering university) and age-matched typical readers across a variety of cognitive and linguistic tasks. All participants completed several tasks, including word and text reading, reading comprehension, lexical decision tasks, auditory rhyming decision tasks, orthographic knowledge, processing speed (sign number test and symbol search), rapid automatized naming (RAN) tests, word fluency tests, working memory digit span tests, and general ability tests. The results showed that, as in Hatcher et al. (2002)’s study, typical readers outperformed dyslexics in almost all reading and reading-related tasks (both in terms of accuracy and speed), except for reading comprehension. Differences between the two groups also extended to other cognitive measures, including working memory and general ability. Furthermore, when comparing the intensity of behavioral effects between adult and child groups, the findings indicated that, in the context of Hebrew’s unpointed orthography, certain deficits observed in child dyslexics were less pronounced in adults (e.g., word reading in context and orthographic ability measures). However, in other tasks, such as phonological processing, the gap between dyslexic and typical readers increased in adults compared to children. In another study conducted on Hebrew-speaking readers (Beidas et al., 2013), the authors compared the cognitive and linguistic profiles of 35 skilled readers and 34 dyslexic students recruited from the student support service for students with learning disabilities at the university of Haifa. All participants underwent a battery of reading-related tasks (decoding accuracy, fluency, reading comprehension) PA, orthographic processing, spelling, speed of processing (RAN and symbol search), and general cognitive abilities. The findings showed that dyslexics not only underperformed controls in decoding and phonology but also demonstrated weaker general cognitive abilities, poorer performance in spelling and visual working memory but also slower naming speed (RAN). A recent study by Faísca et al. (2023) examined a group of 31 dyslexic adults and a group of age-matched controls to assess the heterogeneity among Portuguese-speaking dyslexics. Both groups underwent a battery of cognitive and reading tests that included a reading aloud task, silent reading and sentence completion task, PA tasks, RAN, working memory, and a general ability test. A cluster analysis of the dyslexic participants revealed two distinct profiles. Cluster 1 was characterized by phonological and reading deficits and thus performed below the second cluster in core skills of reading including reading fluency, phonological decoding, alphanumeric RAN, spoonerism, short term memory, reading comprehension, working memory, and nonverbal IQ. In addition, cluster 2 performed below the control group in reading measures, phonological decoding, and reading comprehension but was comparable to the control group in phonological processing measures, visual attention span, and verbal working memory. The authors explained that cluster 2 participants may have compensated for some of their initial phonological deficits through a systematic exposure to reading and writing during childhood. Additionally, their intact cognitive abilities, similar to those of the control group, may have acted as a protective factor, helping them to compensate for reading difficulties. In accord with these results, a new study conducted on 28 highly educated dyslexic, Brazilian Portuguese adult speakers who were diagnosed during childhood (Moojen et al., 2020) revealed that dyslexics performed worse than controls at all levels of phonological awareness, reading, and spelling. The authors highlighted not only the exposure to reading as a compensating factor but also the sensitivity to the structure of texts which can help dyslexic adults better comprehend and engage with academic materials. Eloranta et al. (2019) investigated 48 Finnish adult reading-disabled (diagnosed in childhood) and 37 matched controls to investigate how childhood reading disability predicts dyslexia in adulthood. The study employed a battery of tests to assess reading, cognitive, and linguistic skills in childhood and adulthood. In childhood, children who scored 1.5 standard deviations in reading below the reference group were considered as reading disabled. In adulthood, reading abilities were measured using a Finnish test battery that included word and non-word reading accuracy and fluency, as well as reading comprehension. The participants’ general cognitive abilities were also assessed (using the Wechsler Intelligence Scale for Children and Wechsler Adult Intelligence Scale-IV). Working memory (Digit Span in adulthood), phonological skills, and RAN were administered in both childhood and adulthood. Their results showed that, although globally the reading disability group showed poorer performance than controls in phonological, processing speed, and verbal comprehension skills, over half of the dyslexic individuals improved their reading fluency to a level that no longer met adult dyslexia criteria. Importantly, the study highlighted that in the Finnish shallow orthographic system, childhood rapid naming ability is a significant factor in differentiating individuals with persisting reading disability from those who have significantly improved their reading fluency.

To summarize, the literature suggests that various factors can affect the development of dyslexia in adulthood, resulting in diverse cognitive and linguistic manifestations. However, it is worth noting that to date, almost all the research into adult dyslexics has tended to treat dyslexics as a single undifferentiated group where researchers only reported mean performance, providing thus little information about variability and the possibility of subtypes. In addition, while research on adult dyslexia, particularly in English and other European languages, has increased, there remains a scarcity of evidence-based research in other languages. Given the growing number of dyslexic students in higher education institutions (Dobson Waters & Torgerson, 2021; Pino & Mortari, 2014), understanding the pattern of strengths and weaknesses (i.e., in reading, linguistic and cognitive skills) of this population became a critical issue. Such knowledge can inform evidence-based practices in higher education, enabling psychologists and educators to select appropriate assessment tools and develop targeted interventions and strategies to support the academic journey of adults with reading difficulties. Also, since data generalization from English and other European languages to other non-European and non-alphabetical languages is not straightforward, the current study aims to provide insights into the nature of adult dyslexia among Arabic-speaking university students, with a particular focus on identifying reading subtypes.

Operationally, this study aimed to assess the validity of the accuracy-versus-rate reading subtyping model among Arabic-speaking university students based on specific accuracy and rate achievement criteria using a variety of behavioral measures that have not previously been examined in the context of Arabic. Two questions were addressed:

1. Is there a dissociation between low accuracy and low-rate readers among Arabic-speaking University students?
2. Will there be a difference in the PA and RAN measures among the different reading subtypes?
3. Do reading subtypes show selective pattern of performance on reading and other measures than those used to define them?

Based on previous research in the field and the assumption that literacy abilities/difficulties are conceptualized on a continuum (i.e., typical to mild and more severe/ persistent), we hypothesized that, although the sample examined here is not a clinical one, a dissociation will be observed between students exhibiting low accuracy and those exhibiting low rate together with those who are doubly low readers. In line with previous studies, we expect that such a dissociation will be reflected in the PA and RAN measures.

## Material and methods

### Participants

A total of 120 right-handed adult students, native-speakers of Arabic (age *M* = 22.14, *SD* = 2.38), participated in this study (95 women). All participants were from the University of Haifa or from other nearby academic institutions and studied different disciplines (for more details on the “Material and methods” section, see *Supplementary Materials*).

### Procedure

Experiment flyers were published on social media and within the campus. The recruited students were first questioned to exclude participants with known neurological or psychiatric illness. Those who met the criteria for participating in the study were asked to provide written consent. Individual testing took place in a quiet room and included a battery (~ 2 × 45 min sessions) of reading, linguistic, and cognitive tasks. At the beginning of the session, the participants provided demographic information and filled handedness questionnaire. The study was approved by the Ethics committee of the Faculty of Education.

### Research tools

#### Reading measures for classification

To assess the participants’ reading abilities for the purpose of their classification, a combined measure of reading based on two tests was used as follows:

##### Reading isolated words (Asadi et al., 2014)

Participants were instructed to read a list of 40 words as accurately and fast as possible. Reading accuracy (Cronbach’s alpha = 0.71) and speed were recorded. Reading rate was calculated as the total number of words read per minute, independent of accuracy.

##### Text reading (Asadi et al., 2014)

Participants were asked to read an informational text containing 318 non short-vowelized (referred to as “*non-mashku:l*” text, see Shalhoub-Awwad & Cohen-Mimran, 2024) words, as accurately and fast as possible. Reading accuracy (% of correctly read words) and speed were recorded. Reading rate was computed as the number of words read by minute, independent of accuracy. Combined (words and text) measure for reading accuracy and combined measure for reading rate, used hereafter as the “defining variables for reading subtyping,” was then computed as the average individual accuracy and the average individual rate in the two tests (see details in the “Results” section).

#### Validation measures: reading, linguistic, and cognitive tasks

To examine the validity of reading subtypes, the following reading-related (not used for the subtyping), linguistic, and cognitive tasks were administered to all participants:

##### Non-verbal general ability

General non-verbal ability assessed using the Raven Progressive Matrices test was administered (Raven, 2003).

##### Decoding pseudowords (Asadi et al., 2014)

Participants were asked to read a list of 24 legal pseudowords. Accuracy (Cronbach’s alpha = 0.77) and speed were recorded. Rate was determined as the number of pseudowords read by minute.

##### Reading comprehension

Participants had to silently read a 533-word informational text and to answer 15 multiple-choice questions. Response accuracy was measured. Participants were given 15 min to read and answer the questions related to the text.

##### Phonological awareness

Phonological awareness was assessed using the combined measure of the two following tests:

- Phonemic deletion (Asadi et al., 2014): This 20 item test (for example say: مَريض بلا – م/mari:d̪ˁ/without/m/) examined the students’ ability to delete a phoneme at the beginning, middle and end of words (11, 3, 6 words respectively, Cronbach’s alpha = 0.77). Accuracy was measured.
- Phonemic segmentation (Asadi et al., 2014): The students’ ability to segment words into individual phonemes (for example: جَرَس */jaras*/ = /j/ + /a/ + /r/ + /a/ + /s/) was evaluated using 25 words (Cronbach’s alpha = 0.79). The combined measure of accuracy for phonological awareness (PA) was computed as the average individual accuracy (%) in both tests (*r* = 0.52, *p* < 0.0001).

##### Morphological knowledge

Morphological knowledge was assessed using the combined score of the two following tests:

- Words and verbs inflection (Asadi et al., 2014): This test examined the students’ ability to inflect verbs and nouns in standard Arabic. In this task, participants were asked to produce the verb/noun verbally based on a root/noun, according to gender, tense, and number. This test included 33 items (Cronbach’s alpha = 0.78).
- Morphological fluency task (Asadi et al., 2014): In this task, participants were asked to generate as many as possible words derived from a given three consonants root within 1 min. The combined measure of morphological knowledge was computed as the average individual score for both tests (*r* = 0.32, *p* < 0.001).

##### Rapid automatized naming (RAN)

Two rapid automatized naming (RAN) tests were used: RAN letters and RAN digits. The average time for the two tests (*r* = 0.57, *p* < 0.0001) was used as a combined RAN measure.

##### Working memory

The auditory forward and backward digit span tests (Wechsler, 1998) were administered. A combined measure was then computed as the averaged capacity for the forward and backward tests.

## Results

In order to determine reading subgroups, we relied on previous studies that used a conventional practice based on a 25th percentile low achievement cut-off (Asadi & Shany, 2018; Badian, 1997; Shany & Breznitz, 2011; Shany et al., 2023; Wolf & Bowers, 1999). For this purpose, separate measures of reading accuracy and reading rate were created by combining scores for word and text reading. However, before computing these combined measures, referred hereafter as the “*defining variables for subtyping reading*,” a correlation was computed for each task separately between accuracy and rate and showed in both tasks a positive correlation (*r* = 0.47, *p* < 0.000 for words, and *r* = 0.24, *p* < 0.01 for text). This finding indicated that in both tasks, no tradeoff was found between the two measures of reading. Also, correlations were computed and showed that word reading accuracy correlated positively with text reading accuracy (*r* = 0.64, *p* < 0.000) and that word reading rate correlated positively with text reading rate (*r* = 0.59, *p* < 0.000).

The descriptive statistics for the combined measures are presented in Table 1 (*M Words-Text Acc.* and *M Words-Text Rate*) together with the descriptive statistics for accuracy and rate separately in each task. The combined measures of accuracy and rate showed no tradeoff since they also positively correlated (*r* = 0.41, *p* < 0.000). Finally, the Kolmogorov–Smirnov one-sample test for normality showed a non-significant *d* statistic for the combined accuracy (K-S *d* = 0.109, *p* > 0.15) and for the combined rate (K-S *d* = 0.081, *p* > 0.20) measures, suggesting their distributions were normal.

**Table 1:** Descriptive statistics for reading measures

Measures	Mean (SD)	Range (Max–Min)	Skewness
*Words Acc*	88.3 (8.4)	50.0	− 1.38
*Words Rate*	41.9 (13.2)	66.9	0.45
*Text Acc*	98.4 (1.4)	10.1	− 2.48
*Text Rate*	118.5 (19.5)	119.0	0.9
*M Words-Text Acc*	93.4 (4.7)	29.9	− 1.58
*M Words-Text Rate*	80.23 (14.67)	88.5	0.89

*Acc. (for accuracy)* refers to the percentage of correctly read words in each task, *Rate* refers to the number of words read by minute, *M Words-Text Acc.* refers to combined measure (average words and text) for accuracy, *M Words-Text Rate* refers to combined measure (average words and text) for rate. See the “Results” for the correlations between the different measures

### Is there dissociation between low-accuracy and low-rate readers?

**Fig. 1:**
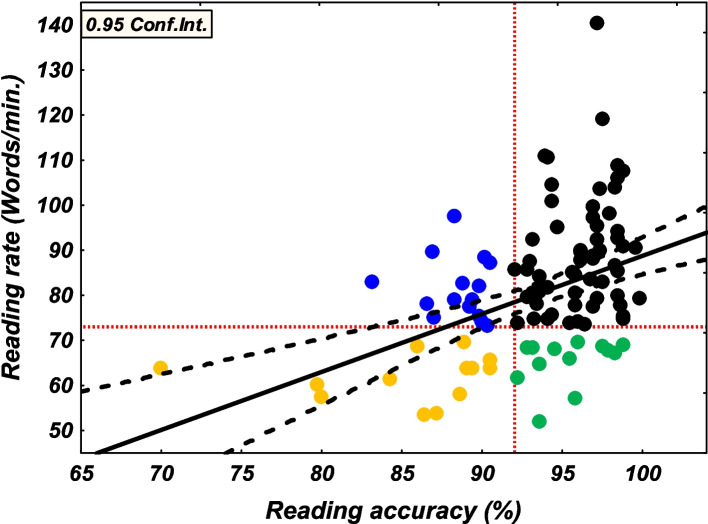
Correlation between accuracy and rate measures (dashed black lines represents the 0.95 confidence interval). Black circles for skilled readers; blue circles for low accuracy; green circles for low rate and yellow circles for doubly low. Horizontal and vertical dashed red lines indicate the 35th percentile value for each measure

Once the two defining variables were computed, subgroups were constituted using the 25th and the 35th percentile cut-offs for each measure. The cut off values for the accuracy measure were = 90.46% and = 92.03% respectively for the 25th and the 35th percentiles. For the rate measure, the cut-off values were = 69.69 and = 73.27 words per minute respectively for the 25th and the 35th percentiles. Based on these values, 12.5% scored ≤ than the 25th percentile in accuracy but ≥ the 35th percentile in rate and was assigned to the low-accuracy group, ~ 10.8% of the readers scored ≤ the 25th percentile in the rate but ≥ the 35th percentile in the accuracy and were assigned to the low-rate group. Finally, those who scored below the 25th percentile in both accuracy and rate measures (~ 10.8%) were assigned to the doubly low group. Figure 1 shows the correlation between the combined accuracy and rate measures and displays the reading subtypes in different colors along the axes of the cut-offs.

Table 2 presents the descriptive statistics for the combined reading measures as a function of the reading groups. A one-sample *t* test (significance level set at *p* < 0.01) revealed significant differences between the mean of each of the three groups and the whole sample mean. As expected, separate one-way ANOVAs with group as a between-subjects factor conducted on accuracy and rate confirmed the existence of significant group effects for both measures. Fisher’s LSD post hoc tests showed that group effect for accuracy was due to higher accuracy in low-rate than in low-accuracy and doubly low groups, the latter two also differing significantly (see statistics in Table 2). As for group effect for rate, the analysis revealed that the low-accuracy group exhibited a significantly higher rate than the two other low groups (low rate and doubly low; see statistics in Table 2) which did not differ significantly, although with doubly low displaying the poorest rate.

**Table 2:** Descriptive statistics (means and SD) for the combined reading measure across the different reading types

	All (*N* = 120)	LA_(A)_ (*N* = 15)	LR_(B)_ (*N* = 13)	DL_(C)_ (*N* = 13)	One-way ANOVA (df 2, 38)	Post hoc
*M Words-Text Acc*	93.3 (4.6)	88.5^a^ (1.9)	95.3^a^ (2.2)	85.3^a^ (5.8)	F = 24.45, *p* = .000, *η*_*p*_^2^ = .56	B > A***, B > C*** A > C*
*M Words-Text Rate*	80.2 (14.6)	81.5 (6.9)	65.3^a^ (5.3)	61.8^a^ (5.1)	F = 45.66, *p* = .000, *η*_*p*_^2^ = .70	A > B***, A > C***

*LA*_*(A)*_ low accuracy, *LR*_*(B)*_ low rate, *DL*_*(C)*_ doubly low (low accuracy and low rate)

Fisher’s LSD post hoc tests: *p < .05; ***p < .001

^a^Comparison of each group subtype (using one-sample t-test) to the whole sample mean indicates significant difference at *p* < .01

### Validation of the reading groups by other measures: do reading subtypes show selective pattern of performance on reading and other measures than those used to define them?

In order to validate the division into the different groups by other measures not included in subtyping, a comparison between the groups and the whole sample means was conducted using the one-sample *t* test (significance level set at *p* < 0.01) for each measure. Additionally, separate one-way ANOVAs were conducted on reading-related and other linguistic and cognitive tasks using group as a between-subjects factor. Table 3 displays the descriptive statistics for all measures in the whole sample and across the three reader subtypes, together with the ANOVAs’ results. The results of these analyses are detailed here below as a function of their appearance in the Table 3.

**Table 3:** Descriptive statistics for the other reading, linguistic, and cognitive measures across the different reading types

Measures	All (*N* = 120)	LA_(A)_ (*N* = 15)	LR_(B)_ (*N* = 13)	DL_(C)_ (*N* = 13)	One-way ANOVA (df 2, 38)	Post hoc tests
*RAVEN*	24.3 (3.3)	25.1 (2.3)	24.3 (3.1)	23.5 (3.4)	*p* = .37	––––
*PW Acc*	78.1 (15.2)	65.0^a^ (13.5)	87.5^a^ (9.8)	60.9^a^ (15.9)	*F* = 15.22, *p* < .000, *η*_*p*_^2^ = .44	B > A***, B > C***
*PW Rate*	34.2 (10.9)	29.4^b^ (7.7)	32.6 (6.8)	23.8^a^ (7.9)	*F* = 4.54, *p* < .05; *η*_*p*_^2^ = .19	B > C**
*Read. Comp*	83.5 (15.2)	82.6 (10.6)	82.3 (12.3)	68.5 (26.1)	*p* = .057	A > C,* B > C*
*Phon. Awar*	76.9 (15.3)	66.4^a^ (9.7)	81.3 (11.3)	53.3^a^ (18.0)	*F* = 14.4, *p* < .000; *η*_*p*_^2^ = .43	B > A**, B > C****, A > C**
*Morph. Know*	67.5 (12.2)	67.7 (11.7)	70.5 (11.2)	56.3^b^ (16.2)	*F* = 4.44, *p* < .05; *η*_*p*_^2^ = .19	A > C,* B > C**
*RAN*	21.0 (3.5)	22.2 (3.1)	22.7 (4.7)	22.3 (3.0)	*p* = *.94*	––––
*Digit Span*	4.7 (0.7)	4.7 (0.5)	4.8 (0.8)	4.7 (0.8)	*p* = .79	––––

*LA* low accuracy, *LR* low rate, *DL* doubly low (low accuracy and low rate), *PW Acc* pseudoword reading accuracy, *Read. Comp.* reading comprehension, *Phon. Awar.* phonological awareness, *Morph. Know.* morphological knowledge, *RAN* rapid automatic naming

Fisher’s LSD post hoc tests: *p < .05; **p < .01; ***p < .001; ****p < .0001

^a^Comparison of each group subtype (using one-sample *t* test) to the whole sample mean indicates significant difference at *p* < .01

^b^Comparison of each group subtype (using one-sample t test) to the whole sample mean indicates significant difference at *p* < .05

#### Raven progressive matrices

The one-sample *t* tests showed no difference between each reader group and the whole sample. Also, the ANOVA conducted on the individual general ability scores showed no significant group effect.

#### Pseudo-word reading accuracy vs rate

The one sample *t* tests conducted on the accuracy measure to compare each group mean score with the whole sample mean indicated that both low-accuracy and doubly low groups performed significantly below the average (with low rate showing a mean accuracy score higher than the average). The one-way ANOVA conducted on this task accuracy measure revealed a highly significant group effect, which to a great extent mimicked that of the combined measure of reading accuracy (see Table 2). Indeed, post hoc tests showed that low-rate group displayed higher pseudo-word reading accuracy than low-accuracy and doubly low readers, the latter two not differing, although low accuracy tended to show higher accuracy than doubly low. As for the rate scores, the one-sample *t* tests showed that low accuracy and doubly low but not low rate differed significantly from the whole group average. The significant group effect in the one-way ANOVA was due to a lowest rate in doubly low group, which significantly differed from low-rate.

#### Reading comprehension

The one sample *t* test showed no differences between the three sub-groups and the whole sample mean. However, the one-way ANOVA conducted on accuracy scores revealed a marginally significant effect of group (*p* = 0.06) due to fact that the doubly low group displayed the lowest accuracy and differed from both the low-accuracy and the low-rate groups.

#### Phonological awareness

The one sample *t* tests for the combined measures of phonological awareness (phonemic deletion: *M* = 85.7, *SD*=14.9% and phonemic segmentation *M* = 68.0, *SD* = 20.1%) showed highly significant differences between both the low-accuracy and doubly low (but not low-rate) groups and the whole sample mean (*p* < 0.01). The one-way ANOVA revealed a highly significant group effect, which perfectly mimicked the effect on the combined measure of reading accuracy (see Table 2), but also the group effect on pseudo-word decoding (Table 3). As Table 3 shows, this effect was due to a higher performance in low rate than in low accuracy and doubly low, but with low accuracy also differing from doubly low.

#### Morphological knowledge

The combined score of morphological knowledge (i.e., morphological fluency *M* = 48.0, *SD* = 17.0% and morphological inflection *M* = 93.1, *SD* = 10.3%) revealed a significant difference only between the doubly low group and the whole sample mean, with no difference between the low-accuracy and the low-rate groups. Additionally, the one-way ANOVA showed a significant group effect. Similar to reading comprehension (see above), Fisher’s LSD post hoc tests showed significantly lower scores in doubly low than in the two other groups which showed no difference.

#### RAN

The combined measure of the rapid automatized naming (RAN) (letters *M* = 24.22, SD = 4.85 s and digits* M* = 17.77, *SD* = 0.31 s) showed no significant differences between the three groups’ mean and the whole sample mean, in addition to the absence of a group effect in the one-way ANOVA.

#### Digit span

The combined measure of the forward and backward sub-tests of this short-term memory test showed no significant differences between the three groups’ mean and the whole sample mean and no group effect in the one-way ANOVA.

## Discussion

The current study aimed to examine the validity of the accuracy versus rate subtyping model (Shany et al., 2023) among Arabic-speaking university students and to assess the specific performance patterns in reading-related and other measures that were not included in the subtyping criteria. To achieve this, a total of 120 Arabic-speaking students were assessed using a battery of reading, linguistic, and cognitive tasks. Separate composite measures of reading accuracy and reading rate, each based on combining scores for word and text reading, were used to define the three subgroups. To classify adult readers into different subgroups, we adopted a common approach in the reading disability literature, using the 25th percentile low achievement cut-offs (Asadi & Shany, 2018; Badian, 1997; Shany & Breznitz, 2011; Shany et al., 2023; Siegel, 1999; Wolf & Bowers, 1999).

The initial key question of the study aimed to determine whether there was dissociation between readers with low accuracy and those with low reading rate. The analysis, based on the combined measures of accuracy and rate, unequivocally demonstrated a clear double dissociation between low achievers in either measure of accuracy (low-accuracy group/LA) or rate (low-rate group/LA), in addition to readers who were concomitantly low achievers in both measures (doubly low/DL). This finding further supports previous research indicating the existence of different types of readers/dyslexics among children (Abu Rabia & Darawshe, 2024; Asadi & Shany, 2018; Gharaibeh et al., 2021; Lovett, 1984a, 1987; Shany & Share, 2011; Wolf & Bowers, 1999) and adults (Cirino et al., 2005; Leinonen et al., 2001; Miller et al., 2006; Shany & Breznitz, 2011; Vukovic et al., 2004; Wolf & Bowers, 1999). Importantly, this observation provides a new validation to Shany and colleagues’ accuracy vs. rate subtyping approach based on word reading (Shany & Breznitz, 2011; Shany & Share, 2011; Shany et al., 2023).

Contrary to other previous studies where subtyping was based on accuracy and rate from a one-word reading task among adults (see for instance Shany & Breznitz, 2011), we opted for separate measures of reading accuracy and reading rate created by combining scores from word- and text-reading tasks. We assumed that a combination of word reading and text reading tasks would better reflect the adult students’ reading abilities. A combined measure for subtyping reading was previously used in Lovett (1984a, 1984b)’s study, where scores from five different tasks were combined to create composite measures of accuracy and rate for both of single-word reading and reading words in context. Although the text-reading task used here might involve additional aspects not present in single-word reading as used by many authors in subtyping studies, we argue that this choice is valid in view of the fact that both the accuracy of the two reading tasks and the rate of the two reading tasks were highly correlated. Besides, the fact that a positive correlation was found between the combined measures for accuracy and also for rate (i.e., the higher is accuracy the higher is rate) ruled out the existence of a trade-off between reading accuracy and rate. Based on both combined measures, our analysis revealed that single-deficit groups (i.e., low-accuracy and low-rate) accounted for quite similar percentages of the sample (with also about the same % for the doubly low group). Importantly, the analysis showed for the first time among Arabic-speaking adult students a dissociation between accuracy-impaired and rate-impaired readers: a group of students exhibited low reading accuracy but with intact reading rate, and another group of students displayed low reading rate alongside intact accuracy. This observation, confirming the existence of distinct subtypes of readers even among university students not diagnosed for dyslexia, is in line with and support previous observations among Hebrew-speaking children (Shany & Share, 2011) and adults (Shany & Breznitz, 2011) and Arabic-speaking children (Shany et al., 2023). These results also provide some support to Lovett’s (1984a, 1984b, 1987) observations among children and Leinonen et al. (2001)’s “*hasty*” and “*hesitant*” subtypes among adults dyslexics.

The distribution of subtypes found here is quite similar to that described by Shany and Share (2011) among Hebrew-speaking fourth graders where each single-deficit group accounted for ~ 10% of the sample (388 children), with ~ 13% of doubly impaired readers. In line with these proportions, Shany et al. (2023)’s analysis of 236 Arabic-speaking fourth graders reported ~ 9.3% of accuracy-disabled, 9.3% of rate-disabled, and 15% of doubly disabled readers. As for the difference in prevalence between ours and Shany and Breznitz (2011)’s results on Hebrew-speaking adult,^1^ it is worth reminding that their analyzed sample included only dyslexics while here, we included adult non-diagnosed students. The observation in their study that the doubly impaired constituted a very small group might probably be explained by the fact that this group is much less likely to enroll in higher education where the academic requirements could be very challenging for them. Doubly impaired readers have been shown to exhibit lower achievements not only in reading tasks but also in various other linguistic and cognitive tasks (Beidas et al., 2013; Faísca et al., 2023; Shany & Breznitz, 2011).

The small differences in prevalence between our sample and the children’s samples of Hebrew (Shany & Share, 2011) and Arabic-speaking (Shany et al., 2023) fourth graders may be attributable to the fact that sampling very young participants better represents the distribution of children with reading difficulties. The fact that children are still in a developmental dynamic where compensation mechanisms are not fully in play might explain the somewhat higher preponderance of doubly impaired. As previously suggested (Reis et al., 2020), results obtained at young ages do probably not provide sufficient information about the long-term constancy of reading difficulties and the actual profile of adults in higher education institutions. This interpretation explains the higher proportion of doubly impaired young readers compared to adult readers, as some of these individuals may develop compensatory strategies over time, leading to a single deficit in adulthood. A recent study showed that among university students diagnosed with dyslexia in childhood (Faísca et al., 2023), some resolve their difficulties along the development, likely due to the systematic exposure to reading and writing. Similar findings were reported by Eloranta et al. (2019) who showed that more than half of Finnish dyslexics had improved their reading fluency to normal adult levels.

The second research question aimed to examine whether reading subtypes would show selective patterns of performance on reading and other measures beyond those used to define the subtypes. Several interesting findings emerged in response to this question. First, it is noteworthy that no differences in general non-verbal ability (Raven test) were found between the reader groups. This suggests that the observed differences in reading performance cannot be explained in terms of cognitive strengths/weaknesses associated with their general intelligence and suggests that students who initially experienced literacy and learning difficulties, but who have managed to reach university level, have developed learning strategies, which helped them to get through and adapt for higher education.

Similar to previous studies in young children and adults, our analysis showed that the doubly low subtype was the most severely impaired group (Asadi & Shany, 2018; Shany & Breznitz, 2011; Shany & Share, 2011; Shany et al., 2023). This group showed low performance, not only in reading accuracy and reading rate, but also in decoding pseudowords (accuracy and rate), reading comprehension, phonological awareness, and morphological knowledge. Our study also demonstrated a differentiation between single-deficit groups in the decoding of pseudowords and phonological awareness accuracy. Specifically, the low-accuracy group performed significantly poorer than the overall sample mean and the low-rate group in these measures. Indeed, both phonological decoding (pseudo-word reading) and phonological awareness measures revealed a pattern of dissociation similar to that observed for the combined measure of reading accuracy, which was considered as the “*defining variable for subtyping reading*.” As for the low-rate group, it clearly did not differ from the whole sample mean (or even showed a higher score) but significantly outperformed the low-accuracy group and, to an even greater extent, the doubly low group. These observations not only support the view of the heterogeneity of dyslexia and confirm the existence of reading subtypes, but also partially support the validity and utility of the accuracy vs. rate classification, both in children (Shany & Share, 2011; Shany et al., 2023) and in adult readers (Leinonen et al., 2001; Shany & Breznitz, 2011).

A distinctive pattern of performance was observed for reading comprehension: no significant differences were found between the single-deficit groups, while both outperformed the doubly low group. This finding is consistent with previous research demonstrating no significant difference in reading comprehension between a Hebrew-speaking dyslexic adult and skilled readers (Beidas et al., 2013; Shany & Breznitz, 2011). However, it contrasts with Shany and Breznitz’s (2011) findings, where reading comprehension differentiated readers’ subtypes: accuracy-disabled and doubly disabled groups underperformed the rate-disabled subgroup, but no difference was observed between the accuracy-disabled and doubly disabled groups. This discrepancy may be attributed to the fact that all participants in the previous study were diagnosed with dyslexia while our sample included “mainstream” university students.

Similar to reading comprehension results, morphological knowledge showed a low performance in doubly low compared to the other two single-deficit groups who performed as well as whole sample. This finding contrasts with Shany and Breznitz (2011)’s findings showing that the rate-disabled group outperformed accuracy-disabled and doubly impaired readers. We postulate that adults having fulfilled the academic requirements had developed compensating mechanisms such as morphological awareness. This assumption aligns with previous research suggesting that dyslexic adults with weak phonological awareness may exhibit strong morphological awareness skills (Cavalli et al., 2017; Law et al., 2015; Leikin & Hagit, 2006).

Contrary to the accuracy of pseudoword decoding and phonemic awareness measures which indisputably showed distinctive patterns of dissociation between single-deficit subgroups, only mixed results were obtained for the decoding rate. Significant differences from the whole sample were observed only for the low-accuracy and doubly low groups, with the latter demonstrating the lowest decoding rate, as previously described. However, contrary to this previous study, we found that the decoding rate of the low-accuracy group differed (i.e., was lower) from that of the whole sample. Although the difference between the two single deficit groups was not significant, this finding aligns with previous observations (Shany & Breznitz, 2011). The absence here of a true dissociation in decoding rate (as in decoding and phonological awareness accuracy) raises the need for some speculations. First, this finding might be explained by the fact that this non-clinical population of high functioning students was not defined as dyslexics and thus do not function as dyslexics. In this particular sample, some individuals (i.e., low-accuracy who clearly have low PA) might be aware of their decoding difficulties and have tended to decode more slowly (i.e., as a compensation mechanism to avoid inaccuracies) and thus their decoding speed resembled that of low rate. Actually, in this specific task where the average words’ frequency equal to zero, the low-accuracy group tended to be even a little slower than the low rate (as in Shany & Breznitz, 2011), probably due to this trade-off (thus disfavoring rate for a “better” accuracy in decoding). This observation fits with other findings on adult dyslexics, often high-functioning university students diagnosed in childhood. These individuals may have benefited from intervention programs, leading to varying degrees of compensation and attenuating the differences between low-accuracy and low-rate readers (Reis et al., 2020). Also, other studies have found similarities between low-accuracy and doubly low groups (Shany et al., 2023). In addition, Lovet (1984, 1987)’s studies among children had previously reported that accuracy-disabled subgroup could also be impaired in reading rate. In Leinonen et al. (2001)’s study among adults, it was reported that the accuracy of the *hesitant* (i.e., slow) subgroup as well as the speed of the *hasty* (i.e., accuracy disabled) subgroup were significantly below normative levels.

Our results showed that RAN measures did not differ significantly between the groups or between sub-groups, and none of the groups differed from the whole sample norm. This result per se contrast with various studies distinguishing typical from dyslexic readers using the RAN measure (Chan et al., 2023; Denckla & Rudel, 1974; Georgiou et al., 2018; Kalindi & Chung, 2018; Valdois et al., 2021) The lack of difference between the subgroups might indicate that RAN might not be a differentiating measure in this non-clinical, high-functioning university students’ group. Similarly, we found no difference between subtypes in verbal short-term memory (i.e., combined digit span). Put together with the observation that the Raven’s general ability test did not differentiate between the sub-groups, these findings suggests that the differences between the three reading subtypes are primarily linked to their reading and linguistic skills, rather than cognitive factors such as general ability, RAN, and short-term memory.

## Conclusions, implications, limitations, and future research perspectives

The findings of the current study suggest that there are distinct reading profiles, characterized by either low reading accuracy, low reading rate, or both. These profiles are associated with partially distinct patterns of reading-related linguistic abilities, with the doubly low subtype exhibiting the lowest performances across most tasks. However, given the fact that some of the validation measures (e.g., pseudo-word reading rate, RAN and MA) showed mixed results, this study only partially supports the accuracy vs rate model (see Shany et al., 2024) of dyslexia in adults and highlight the need for future research to extend these findings to adults who were diagnosed with dyslexia as children. One important implication of this study is that the existence of different subtypes of readers with varying cognitive-linguistic profiles calls for the development of differential intervention programs tailored to these subgroups.

Notwithstanding the relevance of the current study, there are some limitations that should be addressed in future research. One of the difficulties while designing this study was the short in resources of materials in the Arabic language among adults. This fact emphasizes the need for building, improving, and normalizing testing tools for studying such age groups and specific samples. Over and above, despite substantial behavioral research produced in the last years, the neuroanatomical bases of accuracy vs rate model have been little investigated, more specifically among Arabic native-speakers. Future research should also investigate the functional brain correlates of the reading impairments associated to each of the double-deficit model subtypes. Moreover, we used combined reading measure for rate and a combined reading measure for accuracy (both word reading and text reading) as a classifying variable. However, the question remains whether these grouping variables have the same sensitivity in detecting reading subtypes in other languages and if they potentially interact with other linguistic variables, such with orthographic transparency and morphological knowledge. Hence, future studies might extend these results by including other reading-related (e.g., different types of reading comprehension texts), linguistic (e.g., orthographic, syntactic, vocabulary but also morphological), and cognitive measures. As for the sample size, the number of participants in our study presents several limitations that warrant careful considerations. Firstly, the use of cut-offs among a limited sample size may restrict the generalizability of our findings to the broader population of adult university students. Second, the influence of outliers, which may be magnified in such a relatively small sample, could potentially skew our results. These factors, coupled with the challenges of conducting reliable subgroup analysis, necessitate caution in interpreting our findings.

## Supplementary Information

Below is the link to the electronic supplementary material.

Supplementary file1 (DOCX 27 KB)

## Footnotes

1 The sample included 345 University students diagnosed with dyslexia; the accuracy-disabled subgroup included 44.8%, the rate-disabled group included 45.5% of sample and the combined accuracy- and rate-disabled subgroup included 9.7% of the reading-disabled sample.

## References

[CR1] Abu Rabia, S., & Darawshe, E. (2024). Evaluation of the multiple-deficit hypothesis among dyslexic Arabic-speaking children. *Dyslexia,**30*(2), e1759. 38433579 10.1002/dys.1759

[CR2] Asadi, I., Shany, M., Ben-Simon, A., & Ibrahim, R. (2014). لغةُ القراءةِ. *Lorat Elkeraa”*, *the language of reading). A test for the diagnosis of reading and writing disabilities in the Arabic language*, *Based on National Israeli Norms. Yesod Publishers*, *Tel Aviv.(In Arabic)*.

[CR3] Asadi, I. A., Khateb, A., Ibrahim, R., & Taha, H. (2017). How do different cognitive and linguistic variables contribute to reading in Arabic? A cross-sectional study from first to sixth grade. *Reading and Writing,**30*, 1835–1867.

[CR4] Asadi, I. A., & Shany, M. (2018). Examining the double-deficit hypothesis in vowelized–transparent A rabic in a national representative sample of G rades 3 and 4. *Dyslexia,**24*(3), 234–249. 30027673 10.1002/dys.1594

[CR5] Badian, N. A. (1997). Dyslexia and the double deficit hypothesis. *Annals of Dyslexia,**47*, 69–87.

[CR6] Beidas, H., Khateb, A., & Breznitz, Z. (2013). The cognitive profile of adult dyslexics and its relation to their reading abilities. *Reading and Writing,**26*, 1487–1515.

[CR7] Bekebrede, J., van der Leij, A., Plakas, A., Share, D., & Morfidi, E. (2010). Dutch dyslexia in adulthood: Core features and variety. *Scientific Studies of Reading,**14*(2), 183–210.

[CR8] Castles, A., & Coltheart, M. (1993). Varieties of developmental dyslexia. *Cognition,**47*(2), 149–180. 8324999 10.1016/0010-0277(93)90003-e

[CR9] Catts, H. W., Gillispie, M., Leonard, L. B., Kail, R. V., & Miller, C. A. (2002). The role of speed of processing, rapid naming, and phonological awareness in reading achievement. *Journal of Learning Disabilities,**35*(6), 510–525. 10.1177/00222194020350060301 15493249

[CR10] Cavalli, E., Duncan, L. G., Elbro, C., El Ahmadi, A., & Colé, P. (2017). Phonemic—Morphemic dissociation in university students with dyslexia: An index of reading compensation? *Annals of Dyslexia,**67*, 63–84. 27739013 10.1007/s11881-016-0138-y

[CR11] Chan, K., Chung, K. K. H., & Lam, C. B. (2023). What are the cognitive-linguistic profiles and subtypes of Chinese adolescents with dyslexia? *Dyslexia,**29*(4), 369–384. 37528049 10.1002/dys.1748

[CR12] Cirino, P. T., Israelian, M. K., Morris, M. K., & Morris, R. D. (2005). Evaluation of the double-deficit hypothesis in college students referred for learning difficulties. *Journal of Learning Disabilities,**38*(1), 29–43. 15727327 10.1177/00222194050380010301

[CR13] Denckla, M. B., & Rudel, R. (1974). Rapid “automatized” naming of pictured objects, colors, letters and numbers by normal children. *Cortex,**10*(2), 186–202. 4844470 10.1016/s0010-9452(74)80009-2

[CR14] Dobson Waters, S., & Torgerson, C. J. (2021). Dyslexia in higher education: A systematic review of interventions used to promote learning. *Journal of Further and Higher Education,**45*(2), 226–256.

[CR15] Eloranta, A. K., Närhi, V. M., Eklund, K. M., Ahonen, T. P., & Aro, T. I. (2019). Resolving reading disability—Childhood predictors and adult-age outcomes. *Dyslexia,**25*(1), 20–37. 30548736 10.1002/dys.1605

[CR16] Elsherif, M. M., Wheeldon, L. R., & Frisson, S. (2021). Do dyslexia and stuttering share a processing deficit? *Journal of Fluency Disorders,**67*, 105827. 33444937 10.1016/j.jfludis.2020.105827

[CR17] Erbeli, F., Rice, M., & Paracchini, S. (2021). Insights into dyslexia genetics research from the last two decades. *Brain Sciences,**12*(1), 27. 35053771 10.3390/brainsci12010027 PMC8773624

[CR18] Faísca, L., Reis, A., & Araújo, S. (2023). Cognitive subtyping of university students with dyslexia in a semi-transparent orthography: what can weaknesses and strengths tell us about compensation? *Journal of Cultural Cognitive Science,**7*(2), 121–136.

[CR19] Fawcett, A. J., & Nicolson, R. I. (1994). Naming speed in children with dyslexia. *Journal of Learning Disabilities,**27*(10), 641–646. 7844480 10.1177/002221949402701004

[CR20] Fawcett, A. J., Nicolson, R. I., & Dean, P. (1996). Impaired performance of children with dyslexia on a range of cerebellar tasks. *Annals of Dyslexia,**46*, 259–283. 24234275 10.1007/BF02648179

[CR21] Fletcher, J. M., Lyon, G. R., Fuchs, L. S., & Barnes, M. A. (2018). *Learning disabilities: From identification to intervention* (2nd ed.). Guilford Publications.

[CR22] Georgiou, G. K., Ghazyani, R., & Parrila, R. (2018). Are RAN deficits in university students with dyslexia due to defective lexical access, impaired anchoring, or slow articulation? *Annals of Dyslexia,**68*, 85–103. 29511958 10.1007/s11881-018-0156-z

[CR23] Gerber, P. J. (2012). The impact of learning disabilities on adulthood: A review of the evidenced-based literature for research and practice in adult education. *Journal of Learning Disabilities,**45*(1), 31–46. 22064950 10.1177/0022219411426858

[CR24] Gharaibeh, M., Sartawi, A. A., Dodeen, H., & Alzyoudi, M. (2021). Effects of rapid automatized naming and phonological awareness deficits on the reading ability of Arabic-speaking elementary students. *Applied Neuropsychology: Child,**10*(1), 1–13. 30961390 10.1080/21622965.2019.1585247

[CR25] Grigorenko, E. L., Compton, D. L., Fuchs, L. S., Wagner, R. K., Willcutt, E. G., & Fletcher, J. M. (2020). Understanding, educating, and supporting children with specific learning disabilities: 50 years of science and practice. *American Psychologist,**75*(1), 37. 31081650 10.1037/amp0000452 PMC6851403

[CR26] Hatcher, J., Snowling, M. J., & Griffiths, Y. M. (2002). Cognitive assessment of dyslexic students in higher education. *British Journal of Educational Psychology,**72*(1), 119–133. 11916468 10.1348/000709902158801

[CR27] Heim, S., Tschierse, J., Amunts, K., Wilms, M., Vossel, S., Willmes, K., Grabowska, A., & Huber, W. (2008). Cognitive subtypes of dyslexia. *Acta Neurobiologiae Experimentalis,**68*(1), 73. 18389017 10.55782/ane-2008-1674

[CR28] Jorm, A. F., & Share, D. L. (1983). An invited article: Phonological recoding and reading acquisition. *Applied Psycholinguistics,**4*(2), 103–147.

[CR29] Kalindi, S. C., & Chung, K. K. H. (2018). The impact of morphological awareness on word reading and dictation in Chinese early adolescent readers with and without dyslexia. *Frontiers in Psychology,**9*, 511. 29706915 10.3389/fpsyg.2018.00511 PMC5906698

[CR30] Law, J. M., Wouters, J., & Ghesquière, P. (2015). Morphological awareness and its role in compensation in adults with dyslexia. *Dyslexia,**21*(3), 254–272. 25620091 10.1002/dys.1495

[CR31] Leikin, M., & Hagit, E. Z. (2006). Morphological processing in adult dyslexia. *Journal of Psycholinguistic Research,**35*, 471–490. 17082985 10.1007/s10936-006-9025-8

[CR32] Leinonen, S., Müller, K., Leppänen, P. H., Aro, M., Ahonen, T., & Lyytinen, H. (2001). Heterogeneity in adult dyslexic readers: Relating processing skills to the speed and accuracy of oral text reading. *Reading and Writing,**14*, 265–296.

[CR33] Lovett, M. W. (1987). A developmental approach to reading disability: Accuracy and speed criteria of normal and deficient reading skill. *Child Development* (pp. 234-260). 3816346

[CR34] Lovett, M. W. (1984a). A developmental perspective on reading dysfunction: Accuracy and rate criteria in the subtyping of dyslexic children. *Brain and Language,**22*(1), 67–91. 6722529 10.1016/0093-934x(84)90080-4

[CR35] Lovett, M. W. (1984b). The search for subtypes of specific reading disability: Reflections from a cognitive perspective. *Annals of Dyslexia,**34*(1), 153–178. 24243299 10.1007/BF02663618

[CR36] Lyon, G. R., Shaywitz, S. E., & Shaywitz, B. A. (2003). A definition of dyslexia. *Annals of Dyslexia,**53*(1), 1–14. 10.1007/BF02648210 24234186

[CR37] Miller, C. J., Miller, S. R., Bloom, J. S., Jones, L., Lindstrom, W., Craggs, J., Garcia-Barrera, M., Semrud-Clikeman, M., Gilger, J. W., & Hynd, G. W. (2006). Testing the double-deficit hypothesis in an adult sample. *Annals of Dyslexia,**56*, 83–102. 17849209 10.1007/s11881-006-0004-4

[CR38] Miller-Shaul, S. (2005). The characteristics of young and adult dyslexics readers on reading and reading related cognitive tasks as compared to normal readers. *Dyslexia,**11*(2), 132–151. 15918371 10.1002/dys.290

[CR39] Moojen, S. M. P., Gonçalves, H. A., Bassôa, A., Navas, A. L., de Jou, G., & Miguel, E. S. (2020). Adults with dyslexia: How can they achieve academic success despite impairments in basic reading and writing abilities? The role of text structure sensitivity as a compensatory skill. *Annals of Dyslexia,**70*, 115–140. 32221905 10.1007/s11881-020-00195-w

[CR40] Nation, K., & Snowling, M. J. (2004). Beyond phonological skills: Broader language skills contribute to the development of reading. *Journal of Research in Reading,**27*(4), 342–356.

[CR41] Norton, E. S., & Wolf, M. (2012). Rapid automatized naming (RAN) and reading fluency: Implications for understanding and treatment of reading disabilities. *Annual Review of Psychology,**63*, 427–452. 10.1146/annurev-psych-120710-100431 21838545

[CR42] Paap, K. R., & Noel, R. W. (1991). Dual-route models of print to sound: Still a good horse race. *Psychological Research Psychologische Forschung,**53*(1), 13–24.

[CR43] Pammer, K. (2014). Brain mechanisms and reading remediation: More questions than answers. *Scientifica,**2014*, 1–9. 10.1155/2014/802741 PMC3913493 24527259

[CR44] Pennington, B. F. (2006). From single to multiple deficit models of developmental disorders. *Cognition,**101*(2), 385–413. 16844106 10.1016/j.cognition.2006.04.008

[CR45] Pennington, B. F., Cardoso-Martins, C., Green, P. A., & Lefly, D. L. (2001). Comparing the phonological and double deficit hypotheses for developmental dyslexia. *Reading and Writing,**14*, 707–755.

[CR46] Peterson, R. L., & Pennington, B. F. (2012). Developmental dyslexia. *The Lancet,**379*(9830), 1997–2007. 10.1016/S0140-6736(12)60198-6 PMC3465717 22513218

[CR47] Pino, M., & Mortari, L. (2014). The inclusion of students with dyslexia in higher education: A systematic review using narrative synthesis. *Dyslexia,**20*(4), 346–369. 25293652 10.1002/dys.1484 PMC4253321

[CR48] Porpodas, C. D. (2013). Literacy acquisition in Greek: Research review of the role of phonological and cognitive factors. *Handbook of orthography and literacy* (pp. 189 –199). Erlbaum

[CR49] Ramus, F., Rosen, S., Dakin, S. C., Day, B. L., Castellote, J. M., White, S., & Frith, U. (2003). Theories of developmental dyslexia: Insights from a multiple case study of dyslexic adults. *Brain,**126*(4), 841–865. 12615643 10.1093/brain/awg076

[CR50] Raven, J. (2003). *Raven progressive matrices*. Springer.

[CR51] Reis, A., Araújo, S., Morais, I. S., & Faísca, L. (2020). Reading and reading-related skills in adults with dyslexia from different orthographic systems: A review and meta-analysis. *Annals of Dyslexia,**70*, 339–368. 32918699 10.1007/s11881-020-00205-x

[CR52] Roitsch, J., & Watson, S. M. (2019). An overview of dyslexia: Definition, characteristics, assessment, identification, and intervention. *Science Journal of Education,**7*(4), 81.

[CR53] Shalhoub-Awwad, Y., & Cohen-Mimran, R. (2024). On the role of morphology in early spelling in Hebrew and Arabic. *Morphology,**34*(2), 151–172. 10.1007/s11525-023-09408-5 PMC10226023 37361511

[CR54] Shankweiler, D., & Liberman, I. Y. (1989). *Phonology and reading disability: Solving the reading puzzle* (vol. 6). University of Michigan Press

[CR55] Shany, M., Asadi, I., & Share, D. L. (2023). Accuracy-disability versus rate-disability subtypes of dyslexia: A validation study in Arabic. *Scientific Studies of Reading,**27*(2), 136–159.

[CR56] Shany, M., & Breznitz, Z. (2011). Rate-and accuracy-disabled subtype profiles among adults with dyslexia in the Hebrew orthography. *Developmental Neuropsychology,**36*(7), 889–913. 21978011 10.1080/87565641.2011.606410

[CR57] Shany, M., & Share, D. L. (2011). Subtypes of reading disability in a shallow orthography: A double dissociation between accuracy-disabled and rate-disabled readers of Hebrew. *Annals of Dyslexia,**61*, 64–84. 21108026 10.1007/s11881-010-0047-4

[CR58] Share, D. L. (2021). Common misconceptions about the phonological deficit theory of dyslexia. *Brain Sciences,**11*(11), 1510. 34827508 10.3390/brainsci11111510 PMC8615585

[CR59] Share, D. L., & Stanovich, K. E. (1995). Cognitive processing in early reading development: Accommodating individual differences in to a model of acquisition. *Issue in Education: Contributions from Educational Psychology,**1*, 1–57.

[CR60] Shaywitz, B. A., & Shaywitz, S. E. (2020). The American experience: Towards a 21st century definition of dyslexia. *Oxford Review of Education,**46*(4), 454–471.

[CR61] Shaywitz, S. E., & Shaywitz, B. A. (2005). Dyslexia (specific reading disability). *Biological Psychiatry,**57*(11), 1301–1309. 15950002 10.1016/j.biopsych.2005.01.043

[CR62] Siegel, L. S. (1999). Issues in the definition and diagnosis of learning disabilities: A perspective on Guckenberger v. Boston University. *Journal of Learning Disabilities,**32*(4), 304–319. 15508472 10.1177/002221949903200405

[CR63] Snowling, M. J., Hulme, C., & Nation, K. (2020). Defining and understanding dyslexia: Past, present and future. *Oxford Review of Education,**46*(4), 501–513. 32939103 10.1080/03054985.2020.1765756 PMC7455053

[CR64] Sobotka, K. R., & May, J. G. (1977). Visual evoked potentials and reaction time in normal and dyslexic children. *Psychophysiology,**14*(1), 18–24. 834798 10.1111/j.1469-8986.1977.tb01147.x

[CR65] Stanovich, K. E. (2000). *Progress in understanding reading: Scientific foundations and new frontiers*. Guilford Press.

[CR66] Swanson, H. L., & Hsieh, C.-J. (2009). Reading disabilities in adults: A selective meta-analysis of the literature. *Review of Educational Research,**79*(4), 1362–1390.

[CR67] Tannock, R. (2014) *DSM-5 changes in diagnostic criteria for specific learning disabilities (SLD): What are the implications*? International Dyslexia Association.

[CR68] Valdois, S., Reilhac, C., Ginestet, E., & Line Bosse, M. (2021). Varieties of cognitive profiles in poor readers: Evidence for a VAS-impaired subtype. *Journal of Learning Disabilities,**54*(3), 221–233. 32985335 10.1177/0022219420961332

[CR69] Vukovic, R. K., Wilson, A. M., & Nash, K. K. (2004). Naming speed deficits in adults with reading disabilities: A test of the double-deficit hypothesis. *Journal of Learning Disabilities,**37*(5), 440–450. 15460350 10.1177/00222194040370050601

[CR70] Wagner, R. K., & Torgesen, J. K. (1987). The nature of phonological processing and its causal role in the acquisition of reading skills. *Psychological Bulletin,**101*(2), 192.

[CR71] Wagner, R. K., Zirps, F. A., Edwards, A. A., Wood, S. G., Joyner, R. E., Becker, B. J., Liu, G., & Beal, B. (2020). The prevalence of dyslexia: A new approach to its estimation. *Journal of Learning Disabilities,**53*(5), 354–365. 32452713 10.1177/0022219420920377 PMC8183124

[CR72] Wechsler, D. (1998). *Wechsler scales of intelligence-R 95: Hebrew version*. Ministry of Education.

[CR73] Willcutt, E. G., Doyle, A. E., Nigg, J. T., Faraone, S. V., & Pennington, B. F. (2005). Validity of the executive function theory of attention-deficit/hyperactivity disorder: A meta-analytic review. *Biological Psychiatry,**57*(11), 1336–1346. 15950006 10.1016/j.biopsych.2005.02.006

[CR74] Wimmer, H. (1993). Characteristics of developmental dyslexia in a regular writing system. *Applied Psycholinguistics,**14*(1), 1–33.

[CR75] Wolf, M., & Bowers, P. G. (1999). The double-deficit hypothesis for the developmental dyslexias. *Journal of Educational Psychology,**91*(3), 415.

[CR76] Wolf, M., & Bowers, P. G. (2000). Naming-speed processes and developmental reading disabilities: An introduction to the special issue on the double-deficit hypothesis. *Journal of Learning Disabilities,**33*(4), 322–324. 15493094 10.1177/002221940003300404

[CR77] Yang, L., Li, C., Li, X., Zhai, M., An, Q., Zhang, Y., Zhao, J., & Weng, X. (2022). Prevalence of developmental dyslexia in primary school children: A systematic review and meta-analysis. *Brain Sciences,**12*(2), 240. 35204003 10.3390/brainsci12020240 PMC8870220

[CR78] Ziegler, J. C., & Goswami, U. (2005). Reading acquisition, developmental dyslexia, and skilled reading across languages: A psycholinguistic grain size theory. *Psychological Bulletin,**131*(1), 3. 15631549 10.1037/0033-2909.131.1.3

[CR79] Zoubrinetzky, R., Bielle, F., & Valdois, S. (2014). New insights on developmental dyslexia subtypes: Heterogeneity of mixed reading profiles. *PLoS ONE,**9*(6), e99337. 24918441 10.1371/journal.pone.0099337 PMC4053380

