# Supplementary material for: Reading subtyping of Arabic-speaking university students: a contribution to the accuracy vs. rate model of dyslexia
Source: Ann Dyslexia. 2025 Feb 13;75(2):241–61. doi: 10.1007/s11881-025-00323-4 (PMC12238137; doi:10.1007/s11881-025-00323-4)
Supplement: Supplementary file 1 — Supplementary file1 (DOCX 27 KB) [file 11881_2025_323_MOESM1_ESM.docx]

**Supplementary Materials**

**Reading subtyping of Arabic-speaking University students: A contribution to the accuracy vs. rate model of dyslexia**

**Participants**

A total of 120 right-handed (Laterality index *M* = 79.9, *SD* = 28.3, according to the Edinburgh Handedness Inventory) adult students were recruited from different academic institutions and different disciplines (e.g., Education, Psychology, Computer Sciences, etc.)

**Procedure**

All details are text.

**Research tools**

***Reading measures for classification***

To assess the participants’ reading abilities for the purpose of their classification, a combined measure of reading based on two tests was used as follows:

*Reading isolated words (*Asadi et al., 2014*):* This task included 40 words, 12 of which are verbs and 28 are nouns. The number of characters varied between 3-11 letters (average word length *M* = 6.25, *SD* = 1.53) and the number of syllables between 2-6 (Average syllable length *M* = 3.8, *SD* = 0.98(, see examples in Appendix here below.

*Text reading (*Asadi et al., 2014*):* All details in text (**Material and methods**).

***Validation measures: Reading, linguistic and cognitive tasks***

***Non-verbal general ability:*** The Raven Progressive Matrices (RPM) assessed General non-verbal ability (RPM) (Raven, 2003). For the purpose of this study, two 30-items subtests of the RPM were designed by using in one the odd items and in the other the even test items. In each item, the participant was asked to choose from six simultaneously presented alternatives the missing part that completed a matrix. Correct answers were recorded for this task. Half of the participants were administered one subtest and the other half the second subtests (a= .68).

***Decoding pseudo-words*** *(Asadi et al., 2014):* The 25 legal pseudo-words (patterns which answers the rules of Arabic morphological/writing system but with no lexical meaning). The 25 pseudo-words varied from 2-6 characters (average word length *M* = 3.71, *SD* = 1.1) and from 2-4 syllables (average syllable length *M* = 2.79, SD = 0.71, see examples in Appendix).

***Reading comprehension****:* The 533 words text was based on the Psychometric Entrance Test (PET) which is Israel’s SAT tests (October 2012). The 15 questions were composed by the researchers based on the four levels of comprehension (literal comprehension, interpretive comprehension, applied comprehension and affective comprehension) including multiple answers questions. Participants were asked to read the text and answer the questions within 15 minutes. Response accuracy and total time (not used here) were measured.

***Phonological awareness:***

*Phonemic deletion (Asadi et al., 2014):* In this 20 items test, the number of phonemes varied between 3-8 phonemes (*M* = 5.35, *SD* =1.62) and 1-3 syllables (*M* = 2, *SD* = 0.7) and 1-3 syllables (*M* = 2, *SD* = 0.7). Each word was read by the examiner to the participant who had to repeat it and to say it after deleting a specific sound (see examples in Appendix).

*Phonemic segmentation* (Asadi et al., 2014): The 25 items represented 22 words and 3 pseudowords. The number of phonemes varied between 2-6 (*M* =3.88, *SD* =1.18) and the number of syllables varied between 1-2 (*M* =1.44, *SD* =0.49). For each item, the participant had to repeat each word after the examiner and to segment into its individual sounds. Segmentation accuracy and total time were measured (not used here) (see examples in Appendix).

***Morphological knowledge:*** Morphological knowledge was assessed using the combined score of the two following tests:

*Words and verbs inflection (Asadi et al., 2014)*: This test included 33 items. The verb part included 7 roots, each root had a specific gender-number morpheme, and participants were asked to inflect each root- gender- number to three tenses (past, present and imperative), resulting 21 items. In the name inflection part, there were three names which participants were asked to inflect according to four gender-number morphemes (using only possessive pronouns) resulting 12 items.

*Morphological fluency task* (Asadi et al., 2014): Participants were asked to generate as many as possible words derived from a given three consonants (1. */g/ /l/ /s/,2./n/* /*ðˁ/ /f/, 3. /b/ /r/ /d/* ) root within one minute. Number of items for each root was recorded and the total number was calculated. The results were normalized (between 0 and 100) for the total number of productions of each participant.

***Speed of processing:*** This ability was examined based on the combined time of two rapid automatized naming (RAN) tests (Denckla & Rudel, 1974). This test was used to assess naming speed and included 2 subtests consisting each of 50 items (5 items repeated 10 times). The first subtest includes 5 Arabic letters the participants had to name from left to right as fast as possible. The second include 5 Arabic digits the participants had to name from right to left.

***Working memory:*** The forward and backward digit span tests (Wechsler, 1998) assessed participants’ short term working memory. Participants were asked to repeat a series of digits after hearing them either in the same order of in the reverse order. The maximal level to which the participants reached was determined for each sub-test.

**Appendix**

Examples of different stimulus in the different tasks with phonetic translation

| Example Task | Example 1 | Example 2 | Example 3 |
| --- | --- | --- | --- |
| *Reading isolated words* | شُموخ  /ʃumu: x / | يَفْزعونَ  / yafzaʕu:na / | اِستَسقى  /ʔistasqa:/ |
| *Decoding pseudo-words* | تَشَفُ  /taʃafu/ | لُعُ  /luʕu/ | بَراشِفُ  /bara: ʃ ifu/ |
| *Phonemic deletion* | مَريض بلا – م  /mari:d̪ˁ / without /m / | رُز بلا -ز  / ruz / without /z/ | مسْطَرَه  بلا -ط  /mas t̪ˁara /without /t̪ˁ/ |
| *Phonemic segmentation* | جَرَس  /garas/ | زام  /za:m/ | شَمْس  /ʃams/ |
| *Words and verbs inflection* | غ.ن.ى (هم-ماضي)  / ɣ .n.a: (hum-in past)/  غنّوا- they sang  / ɣannaw / | ل.ب.س (هن-مضارع)  /l.b.s (hun- in present)/  يلبسنَّ-they wear  / yalbasna / | حاسوب (هو) /‎ ħasu:b (huwa)/  حاسوبهُ- his laptop  / ħa:su:bahu / |
| *Morphological fluency task* | ج.ل.س- sit  /ʒ.l.s / | ن.ظ.ف- clean  /n. ðˁ.f / | ب.ر.د-cold  /b.r.d / |
